# Effect of pharmacist intervention on physician prescribing in patients with chronic schizophrenia: a descriptive pre/post study

**DOI:** 10.1186/s12913-016-1408-4

**Published:** 2016-04-26

**Authors:** Yasuhiko Hashimoto, Masami Tensho

**Affiliations:** Faculty of Clinical Pharmacy, School of Pharmaceutical Science, Kobe Gakuin University, 1-1-3 Minatojima, Chuo-ku, Kobe, 650-8586 Japan; Department of Pharmacy, Sawa Hospital, 1-9-1 Shiroyama-cho, Toyonaka, Osaka 561-0803 Japan

**Keywords:** Medication cost, Pharmacist intervention, Pharmacist, Schizophrenia

## Abstract

**Background:**

Although pharmacotherapy is one of the most important treatments for schizophrenia, the prominent levels of antipsychotic polypharmacy and high-dose regimens used in Japan are thought to be inconsistent with treatment regimens used in other countries. In this study, we evaluated the effect of pharmacist intervention on physician prescribing in patients with chronic schizophrenia.

**Methods:**

Participants comprised 52 inpatients at Sawa Hospital (Osaka, Japan), treated with at least one antipsychotic agent, who received pharmacist intervention for 1 year (2012). We compared the dose and the number of antipsychotics prescribed, and the rate of concurrent prescribing of anti-Parkinson, benzodiazepine and mood-stabilizer medication, pre- and post-pharmacist intervention. As an indicator of psychosis symptoms, the rate of seclusion room use was recorded. Additionally, we evaluated the impact of pharmacist intervention on medicine costs. Continuous variables were analyzed by Wilcoxon signed-rank sum tests, and categorical data were analyzed using Fisher’s exact tests.

**Results:**

Compared with pre-intervention, the dose (982.6 mg pre vs. 857.6 mg post; *p* < 0.01) and the number of antipsychotics (2.0 pre vs. 2.0 post; *p* < 0.05) at 1 year were significantly lower post-intervention. The seclusion room use rate was not significantly different but tended to be lower post-intervention than pre-intervention (*p* < 0.1). The cost (in USD) for all medicines (10.33 pre vs. 8.76 post; *p* < 0.05), antipsychotics (8.04 pre vs. 6.48 post; *p* < 0.05), and psychotropics (9.24 pre vs. 7.68 post; *p* < 0.01) were significantly lower post-intervention than pre-intervention.

**Conclusion:**

Pharmacist intervention has the potential to optimize medication prescribing and reduce medication costs in patients with chronic schizophrenia. It might be suggested that clinical practitioners as well as medical hospital administrators consider the pharmacists’ ability to rationalize medication therapy in schizophrenia.

## Background

Despite the poor and contradictory evidence for the effectiveness of two or more antipsychotics (antipsychotic polypharmacy), it continues to be used in about one third of patients with chronic schizophrenia [1]. Disadvantages of polypharmacy include the potential for increased adverse effects [2, 3] and increased healthcare costs [4]. Often when managing these adverse effects, rather than stopping the offending medication, physicians tend to add a new medication to counteract the adverse effect [5–7]. For example, to manage the adverse effect of extrapyramidal symptoms, an anti-Parkinson medication is added, which causes constipation and a decline in cognitive function. Consequently, patients are prescribed laxatives, and cognitive impairment, an important negative symptom of schizophrenia, is exacerbated [8, 9]. This continued cycle warrants improved physician understanding of the use and effectiveness of medication, and how to better manage adverse effects.

Traditionally the role of the pharmacist predominantly involved the dispensing of medications in hospital pharmacies, and the pharmacist was quite detached from other healthcare professionals [10]. In Japan, although pharmaceutical care by clinical pharmacists was initiated in 1989, this care only guided the use of prescribed medicines. In 2010, the Japanese Ministry of Health, Labour, and Welfare recommended including pharmacists as part of a multidisciplinary team because pharmacists are experts on medicines and are responsible for pharmacotherapy. In these recommendations, clinical pharmacists are asked to optimize prescriptions for patients, taking into account the safety of the pharmacotherapy, especially for drugs treating psychosis. In countries outside Japan, the role of the clinical pharmacist had already evolved and had been recognized as an essential component of the multidisciplinary team [11]. These clinical pharmacists have diversified into expanded areas of care in hospital practice [12], not only checking prescriptions but also discussing cases with physicians, nurses, and other healthcare professionals involved in the care of the patient as well as proposing optimal therapy. These interventions are integral components of the new, enhanced role of the pharmacist in the clinical setting, and if undertaken judiciously, may prevent errors in prescribing [13, 14].

In this study, we evaluated the usefulness of pharmacist intervention on physician prescribing in patients with chronic schizophrenia.

## Methods

### Participants and setting

Fifty-two inpatients at Sawa Hospital (Osaka, Japan) were recruited between October 2010 and October 2012. Patients had a diagnosis of schizophrenia according to the Diagnostic and Statistical Manual of Mental Disorder IV classification and were receiving at least one antipsychotic agent. Their symptoms and medication were stable for at least 3 months as determined by their physicians and by pharmacists examining the patients’ medical histories before inclusion in this study. Exclusion criteria included the presence of symptoms or severe adverse effects that warranted an immediate medication change.

This was a two-stage (pre-/post-intervention) study. In the pre-intervention stage (from 1/11/2010 to 31/10/2011), patients received usual care (no pharmacist intervention). In the post-intervention stage (from 1/11/2011 to 31/10/2012), patients received pharmacist intervention in addition to usual care.

### Pharmacist intervention

For the purposes of the study, the components of pharmacist intervention to optimize and simplify a prescription were outlined as follows. 1) Discuss the decision of polypharmacy and/or excessive antipsychotic doses (defined as more than 1000 mg/day chlorpromazine equivalents). 2) In the discontinuation of antipsychotic medication, propose gradual tapering. 3) Suggest the addition or discontinuation of concurrent medications. 4) Where applicable, recommend therapeutic monitoring to monitor adverse effects. There was no limit to the frequency of intervention provision, to reflect the real clinical setting where interventions are undertaken when deemed appropriate and not at specified intervals.

### Data collection

We recorded the dose and the number of antipsychotics, the rate of concurrent anti-Parkinson, benzodiazepine, and mood-stabilizer use and medicine costs. Doses of antipsychotics were expressed as chlorpromazine equivalents [15]. For patients receiving two or more antipsychotics, the total combined dose was calculated. Data recorded for the pre-stage (at 31/10/2011) was compared with the post-stage (at 31/10/2012), and the rate of seclusion room use was also documented.

### Data analyses

All analyses were carried out using statistical application SPSS (version 19.0, IBM, Japan). The rate of concurrent anti-Parkinson, benzodiazepine and mood stabilizer use and seclusion room use were analyzed using Fisher’s exact tests. The medicine costs and the dose and number of antipsychotics were evaluated using Wilcoxon signed-rank test. The medicine costs, calculated as cost per day, were recorded in USD ($1 = 123 Japanese Yen ). *P* < 0.05 was considered statistically significant.

## Results

### Participant characteristics

A total of 63 patients participated in this study, with 53.9 % of them being men. Among the participants, ten patients had severe symptoms and/or adverse events. One patient was discharged from the hospital before completing the study. Thus, 52 participants completed the study. The average age and illness duration of the participants were 51.6 (SD, 16.8) years and 264.9 (SD, 183.4) months, respectively.

### Effect on physician prescribing, rate of seclusion room use, and medicine costs

As shown in Table 1, the dose and the number of antipsychotic agents were significantly reduced in the post-intervention group as compared with the pre-intervention group (*P* < 0.001 and *P* < 0.05, respectively). The prevalence of as a proportion of antipsychotic polypharmacy in pre- and post-intervention was 73.1 and 63.5 %, respectively. Although there were no significant differences in the rate of concurrent medication use, there was a trend towards lower seclusion room use in the post-intervention group (*P* = 0.077). The cost (in USD) per day of all medicine was significantly reduced from a median of $10.33 pre-intervention to $8.76 post-intervention (*P* < 0.05). Additionally, the costs of antipsychotics from $8.04 to $6.48 (*P* < 0.05), and psychotropic agents, from $9.42 to $7.68 (*P* < 0.005), were also significantly reduced.

**Table 1:** Effects of pharmacist intervention on physician prescribing, frequency of seclusion use, and medicine costs

	Pre-intervention in 2011 (*n* = 52)	Post-intervention in 2012 (*n* = 52)	*p*-value
Antipsychotic			
dose (mg)	982.6 (200.0–2395.1)	857.6 (75.0–2418.6)	*p* < 0.001
No. of medicine(s)	2.0 (1.0–6.0)	2.0 (1.0–5.0)	*p* = 0.025
Concurrent agent n (%)			
anti-Parkinson	25 (48.1)	24 (46.2)	*p* = 0.500
benzodiazepine	42 (80.8)	38 (73.1)	*p* = 0.243
mood stabilizer	28 (53.8)	30 (57.5)	*p* = 0.423
Seclusion room use n (%)	23 (44.2)	15 (28.8)	*p* = 0.077
Cost (USD)			
all medicine	10.33 (1.33–104.79)	8.76 (0.45–34.16)	*p* = 0.045
antipsychotic	8.04 (0.25–32.47)	6.48 (0.25–33.66)	*p* = 0.016
psychotropic	9.42 (0.25–64.12)	7.68 (0.25–34.05)	*p* = 0.004

Continuous variables indicate the median, and range in parentheses

## Discussion

Despite the paucity of data, antipsychotic polypharmacy remains a common practice [16], with an estimated use among individuals with schizophrenia ranging from 10 to 30 % [1].

In this study, we evaluated the effect of pharmacist intervention on physician prescribing. In comparison with the pre-intervention group, the dose and number of antipsychotics in the post-intervention group were significantly lower. With regards to psychotic symptoms, as measured by the rate of seclusion room use, there was a downward trend. Algorithms and guidelines recommend antipsychotic monotherapy as the preferred option [17, 18], and it is generally accepted that the optimal dose of antipsychotics for schizophrenia is 300–800 mg per day chlorpromazine equivalent [19]. In this study, the doses of antipsychotics pre-intervention were beyond these criteria in many patients. Previous studies have shown that reducing the dose and the number of antipsychotic agents increased the Global Assessment of Functioning value significantly [20, 21]. Extrapolating this finding to our results, we speculate that reducing the dose of antipsychotic agents may have been related to the improvement in symptoms. It has been suggested that the high doses of antipsychotics together with polypharmacy, often undertaken by psychiatrists, could adversely complicate medication therapy.

In this study, seclusion room use was used as an index of symptomatic psychosis, but many studies use the Positive and Negative Syndrome Scale (PANSS) and Brief Psychiatric Rating Scale (BPRS) to measure psychiatric symptoms of schizophrenia [22]. However, these scores are based on physician subjectivity, unlike the seclusion room use, which is based on both physicians’ and other healthcare professionals’ opinions. Previous reports also cite seclusion room use as an index of symptomatic schizophrenia [23], yielding similar results to our study, in which the intervention group is more effective than the non-intervention group. A possible explanation for this trend is that all antipsychotics possess a dopamine-2 receptor antagonistic effect, which is the mechanism for suppression of symptoms of schizophrenia. However, excessive antagonism with large antipsychotic doses and polypharmacy results in up-regulation and super sensitivity at the receptor level [24]. Therefore, psychosis might be exacerbated despite compliance with the medication. To manage the acute phase of psychosis, many psychiatrists often have a tendency to add multiple antipsychotics resulting in polypharmacy [25]. This provides only transient relief of symptoms, and physicians remain unsure of which medicines are effective and are consequently reluctant to remove any antipsychotics [26]. The resultant effect is excessive sedation. Therefore, to avoid undesirable adverse effects such as sedation when treating acute symptoms of psychosis, physicians need to prescribe optimal dose of antipsychotics. If symptoms remain and are treatment-resistant, rather than introduce additional agents, or increase the dose of existing antipsychotics, physicians should prescribe agents with a different mechanism of action (such as benzodiazepine or mood stabilizer) for a brief period. A previous study showed that augmentation of antipsychotics with valproic acid, a mood stabilizer, was useful in patients with schizophrenia [27]. With this approach, the excessive antagonistic action against dopamine-2 receptors is avoided. Our study found that, despite a reduction in the number of antipsychotics prescribed, there was no difference in concurrent benzodiazepine and mood stabilizer use between the pre-intervention and post-intervention groups. The reason for this might be in our strategy for tapering the antipsychotics. For pharmaceutical intervention, benzodiazepines and mood stabilizers were first discontinued. After confirming any changes in psychological symptoms, antipsychotics were then gradually tapered off. However, if symptoms became exacerbated, physicians were not allowed to prescribe antipsychotics but were requested to reinstate a benzodiazepine and/or mood stabilizer. Thus, concurrent use of benzodiazepine and mood stabilizers was not reduced in this study.

Regarding medicine costs, few data are available, particularly studies reporting cost–benefit of pharmacist intervention in Japanese hospitals. The cost per patient per day of all medicines, antipsychotic medicines, and psychotropic agents were significantly lower post-intervention compared with pre-intervention. A cost reduction of $1.60 per day extrapolates to a cost reduction of $584.00 per year. As described above, to optimize the antipsychotics, we recommended that physicians use adjunctive benzodiazepine and/or mood stabilizer. However, in this study, the cost of psychotropic agents was also lower, despite no changes in the co-administration ratio. This suggests that another medicine was removed, such as laxatives for constipation.

Given the current estimation of 125,000 patients receiving antipsychotic polypharmacy [28], it would be easy to extrapolate that pharmacist intervention would lead to large economic savings.

There were several limitations to this study. First, because this study was a pre/post design, no concurrent control group was included as each patient served as his or her own control. Therefore, there was no selection bias. As for confounders, the participants were all inpatients and thus received no other therapy. However, natural healing over time could be considered a confounding factor. Second, as the targeted hospital was only one site, the number of participants was low. Third, although it is common for physicians to assess symptoms of schizophrenia using PANSS and BPRS, we evaluated the symptoms/severity using only the measure of seclusion. To fully elucidate the schizophrenia symptoms/severity, the subjective and objective opinions of physicians and other healthcare professionals would be needed. Finally, considering that we excluded ten patients from pharmaceutical intervention owing to any cause, pharmaceutical intervention might be limited to patients who were sufficiently stable. Therefore, to accurately determine the contribution of clinical pharmacists to physician prescribing, a multi-center large-scale randomized study would be needed.

## Conclusion

In this study, we evaluated the usefulness of clinical pharmacist intervention on physician prescribing. The dose and the number of antipsychotics were significantly reduced without an increase in concurrent benzodiazepine and mood stabilizer use following pharmacist intervention. Additionally, seclusion room use tended to be lower and a significant reduction was observed in medicine costs. This suggests that including a clinical pharmacist as one of the multidisciplinary team contributes not only to optimizing physician prescribing but also to the healthcare economy.

### Ethics approval and consent to participate

This study was approved by the Ethics Committee of Sawa Hospital. All patients provided written informed consent to participate in the study. Data accruing was kept securely locked at all times and was only accessible to researchers.

### Availability of data and materials

The data supporting the conclusions of this article are available upon request from the corresponding author.

## Abbreviations

BPRS: brief psychiatric rating scale
PANSS: positive and negative syndrome scale
USD: U.S. dollar
